# Effect of Oral Alpha Lipoic Acid in Preventing the Genesis of Canine Diabetic Cataract: A Preliminary Study

**DOI:** 10.3390/vetsci4010018

**Published:** 2017-03-16

**Authors:** David L. Williams

**Affiliations:** Department of Veterinary Medicine, University of Cambridge, Madingley Road, Cambridge CB3 0ES, UK; dlw33@cam.ac.uk

**Keywords:** diabetes, canine, cataract, lens, alpha lipoic acid, aldose reductase

## Abstract

Blinding cataract is a significant effect of canine diabetes with 75% of animals affected two years after diagnosis. Lens opacification occurs primarily through the generation of sorbitol, a sugar alcohol, through the action of aldose reductase (AR). The osmotic effect of sorbitol draws water into the lens, causing opacification. Inhibition of AR should thus prevent the generation of cataracts. A topical AR inhibitor has been shown to have this effect, as has the commercially available neutraceutical OcuGLO, containing the AR inhibitor alpha lipoic acid (ALA) together with other plant-based antioxidants. Here a comparison is made between the number of diabetic dogs developing cataracts when given oral ALA alone and those given a mix containing ascorbic acid and tocopherol. Animals given ALA developed significantly fewer lens opacities than those given conventional antioxidants. Cataracts which formed occurred at a significantly greater duration after the commencement of treatment than those on the antioxidant mix. Although this is a small study conducted over a short period, the significant benefit of ALA in diabetic dogs is a reason to evaluate these effects in larger trials. As AR is involved in diabetic retinopathy and neuropathy, this enzyme inhibitor may be worthy of evaluation in preventing these conditions in human diabetics also.

## 1. Introduction

The development of blinding lens opacification is widely recognised as a significant problem in diabetic dogs [1]. The opacity appears rapidly as glucose in the lens is metabolised to sorbitol, the sugar alcohol of glucose [2]. The normal lenticular metabolism of glucose to carbon dioxide and water occurs predominantly through anaerobic metabolism, given both the avascular nature of the lens and its lack of mitochondria in all but the lens epithelium. The rate limiting step in glucose metabolism in the lens is phosphorylation of glucose to glucose 6 phosphate by the enzyme hexokinase [3,4]. However, when glucose in the lens reaches a concentration where the enzyme hexokinase is saturated, another enzyme, aldose reductase, can convert the glucose into sorbitol which has a higher osmotic potential than does glucose. This osmotic gradient draws water into the lens, resulting in a rapid development of cataracts [4]. This generation of lens opacity is generally considered as a rapid event, developing from a clear lens to a mature cataract in a matter of hours or days, generally occurring bilaterally symmetrically. It may be that this is not necessarily the case since veterinary ophthalmologists see diabetic dogs at the point when they have become blind as the second eye progresses from a non-blinding cataract to a mature cataract. In fact, initial changes in lens opacity prior to the development of a mature cataract may occur in diabetic dogs with equatorial vacuoles and cortical opacities occurring in a substantial number of cases, and not necessarily occurring bilaterally symmetrically. Similar changes are seen in dogs experimentally fed galactose [5] and other biochemical events occur in the lenses of diabetic dogs prior to this rapid development of a mature cataract through osmotic effects. Thus, equatorial vacuoles and cortical opacities occur in all diabetic dogs even before blinding cataracts, and these do not necessarily occur bilaterally symmetrically. Two other mechanisms are important in the generation of diabetic lens opacification: those involving non-enzymatic glycation of lens proteins [6] and oxidative stress [7]. Aldose reductase and glutathione reductase compete for NADPH in the diabetic lens, resulting in depletion of glutathione (GSH) which is a key antioxidant molecule within the lens, although the part played by these mechanisms in the canine lens is, at present, unclear. 

It has already been demonstrated that topical administration of the aldose reductase inhibitor Kinostat inhibits the development of cataracts in diabetic dogs [8,9], although that product is as of yet unavailable commercially. Similar effects have been achieved with oral administration of a mixture of the aldose reductase inhibitor alpha lipoic acid together with a number of antioxidants available as the neutraceutical OcuGLO^TM^ [10]. Here we seek to show that the aldose reductase inhibitor alpha lipoic acid given on its own has a similarly beneficial effect in slowing the formation of diabetic cataracts in the dog. 

**Figure 1:**
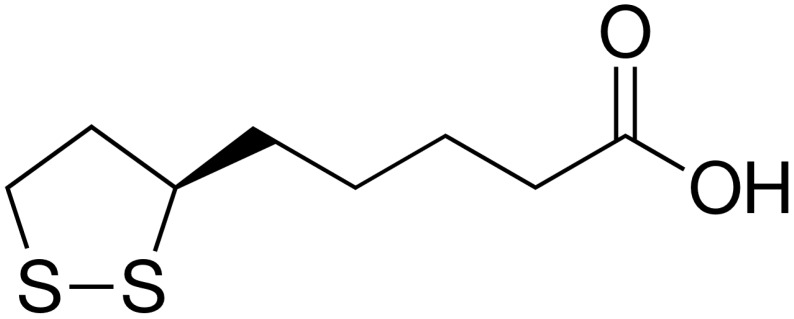
Alpha lipoic acid showing the disulphide bridge existing in the reduced form.

Alpha lipoic acid (Figure 1) has been shown to be safe as an oral preparation in the dog [11] and is a potent antioxidant, a powerful metal chelator and a scavenger of hydroxyl radicals, hypoclorous acid and singlet oxygen species. It also functions to regenerate glutathione to its reduced form [12]. Its reduced form, dihydrolipoic acid, acts as a scavenger of superoxide and a potent inhibitor of lipid peroxidation. Alpha lipoic acid has been shown to have beneficial effects in several models of diabetes, including the streptozocin-induced diabetic cataract in rats [13], and protects of retinal ganglion cells in diabetic retinopathy [14].

## 2. Materials and Methods

Thirty diabetic dogs accessed through the author’s ambulatory ophthalmology referral clinic to 25 first opinion veterinary centres were enrolled in the study prior to developing blinding diabetic cataract. All animals underwent a full ophthalmic examination using direct and indirect ophthalmoscopy and slit lamp biomicroscopy after pharmacological mydriasis. Full informed consent was obtained from the animals’ owners and all animals were treated in accordance with the guidelines for animal use of the Association for Research in Vision and Ophthalmology available at http://www.arvo.org/About_ARVO/Policies/Statement_for_the_Use_of_Animals_in_Ophthalmic_and_Visual_Research/. The study was evaluated and approved by the Ethics and Welfare Committee of the Queens Veterinary School Hospital, University of Cambridge. The dogs were randomly assigned to the treatment group given alpha lipoic acid per os at a dose rate of 2 mg/kg or a placebo group receiving a per os antioxidant mix of ascorbic acid (5 mg/kg per day) and tocopherol (2 iu/kg per day), as used in the study comparing OcuGLO and placebo [10] Dogs were treated continually as anecdotal evidence with the OcuGLO suggested that stopping use of the medication led to cataract formation within a few weeks. The diabetic status of the animals was assessed using the data from blood glucose curves and serum fructosamine levels as documented by the clinic form which the animal was accessed. Animals were examined at monthly intervals and any development of lens opacity was deemed to represent a treatment failure within the study protocol. Time to development of lens opacity on alpha lipoic acid and placebo was compared by means of the Kaplan Meier survival analysis using an SPSS statistics package (IBM United Kingdom Limited, Portsmouth, PO6 3AU, UK).

## 3. Results

**Figure 2:**
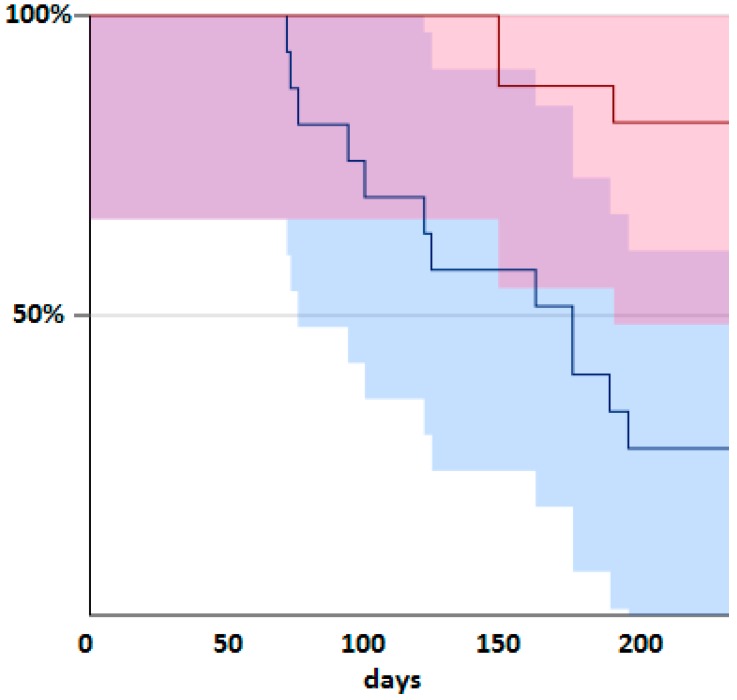
Kaplan Meier plot of time cataract-free on alpha lipoic acid (red) or placebo (blue).

The signalment of the 30 dogs, their serum fructosamine level indicating the degree of diabetic control and the duration in days cataract-free is documented in Table S1. Kaplan Meier plots of the time to the development of lens opacification are shown in Figure 2. The mean ages of the dogs were 10.2 ± 1.5 years in the placebo group and 9.1 ± 1.2 years in the alpha lipoic acid group, these two not being statistically significantly different (*p* = 0.12). Their fructosamine levels were 514 ± 123 μmol/L in the placebo group and 504 ± 126 μmol/L in the alpha lipoic acid group, these figures again not significantly different between the two groups (*p* = 0.11). The duration without significant cataract development was 290 ± 51 days in the alpha lipoic acid group and 162 ± 47 days in the placebo group, these being significantly different (*p* < 0.001). The days to cataract development in eight animals on the placebo treatment that developed lens opacity was 148 ± 9, while for those three on the alpha lipoic acid that developed cataracts, the mean time to cataract formation was 240 ± 36 days, with these significantly different at *p* < 0.01. The Kaplan Meier plot in Figure 2 shows the difference in time to cataract development graphically. 

## 4. Discussion

Blinding cataract is a significant effect of diabetes in the dog, with substantial numbers of animals requiring cataract surgery after developing the mature lens opacity seen in diabetic dogs [1]. A retrospective study has shown that half of the population of diabetic dogs studied had developed cataracts by the 170th day after diagnosis, while 75% developed cataracts by 370 days [15]. The placebo group here exhibited a similar time to development, with the average time to cataract formation being a little over 160 days in the eight dogs affected, while in the three of the dogs treated with alpha lipoic acid per os that developed cataracts, their average time to lens opacification was 80 days longer. The dogs on alpha lipoic acid that did not develop cataracts remained with clear lenses for an average of 395 ± 104 days and were still visual when the study was concluded, and they are still taking alpha lipoic acid daily. Alpha lipoic acid has been shown both in biochemical structure-function studies [16] and therapeutically in a number of studies in laboratory rodents and human patients [17,18,19,20,21,22] to inhibit aldose reductase. The dose rate used here of 2 mg/kg daily was chosen as this was the dose of alpha lipoic acid in OcuGLO, previously shown to inhibit the formation of diabetic cataracts [10] and well within the safe levels of the compound as previously determined [11]. While it would have been interesting to evaluate the serum levels of alpha lipoic acid in the dogs while treated, taking a blood sample for this reason and not directly for the benefit of the animal would be contrary to the Veterinary Surgeons Act (1966) and would have required the animals to be placed under the Animals (Scientific Procedures) Act 1986, which, as the study subjects were pet and not laboratory animals, was not possible under UK legislation. Clearly the results reported here must remain preliminary and further investigation is necessary to increase the number of animals treated and to lengthen the time over which the animals are examined. Nevertheless, the statistically significant difference in time remaining cataract-free while being prescribed alpha lipoic acid suggests that this readily available antioxidant and aldose reductase inhibitor should be used in all diabetic dogs to prevent or, at the very least, delay the onset of blinding cataracts in diabetic dogs. 

The delay in cataract formation in diabetic dogs taking alpha lipoic acid is important not just for those animals, but also as a potential model for aldose reductase–associated diabetic complications in man. We know that polyol formation is important in pericyte loss and endothelial proliferation in diabetic retinopathy in human patients [21], so a readily available oral supplement which reduces such changes would be more than welcome given the large number of patients potentially blinded by such vascular changes. The metal chelating activity of alpha lipoic acid may account for much of its aldose reductase action and clinical effects [22]. Polyol-related cataractogenesis is much more severe in diabetic dogs than in species with lower lenticular aldose reductase activity such as cats [23] and mice [24,25], but it is more severe in rats with a higher level of lens aldose reductase [22]. Such intraspecific differences, together with the fact that mice with genetically engineered elevations in their lens aldose reductase have increased diabetic cataract formation [25], support the concept that it is this enzyme and its effects which are the predominant cause of the readily developing lens opacity in dogs. The ability to prevent such changes using a relatively inexpensive oral supplement such as alpha lipoic acid could significantly change the morbidity of diabetes in pet dogs where cataract development is a significant problem and in human patients with aldose reductase-related diabetic complications. 

## 5. Conclusions

Here we have shown that alpha lipoic acid, potentially acting as both an anti-oxidant and aldose redctase inhibitor, delays and possibly prevents the onset of cataract in diabetic dogs. Given that lens opacification is a significant side effect of diabetes in this species, it is hoped that this molecule can be used more widely to prevent cataract formation in dogs affected by diabetes mellitus and potentially humans with aldose-reductase-associated diabetic pathology such as diabetic retinopathy. 

## Supplementary Materials

The following are available online at www.mdpi.com/2306-7381/4/1/18/s1, Table S1: Signalment of dogs, serum fructosamine and duration cataract-free.
