# Supplementary material for: Effect of Oral Alpha Lipoic Acid in Preventing the Genesis of Canine Diabetic Cataract: A Preliminary Study
Source: Vet Sci. 2017 Mar 16;4(1):18. doi: 10.3390/vetsci4010018 (PMC5606614; doi:10.3390/vetsci4010018)
Supplement: Supplementary file 1 [file vetsci-04-00018-s001.pdf]

Supplementary Materials: Effect of Oral Alpha Lipoic Acid in Preventing the Genesis of Canine Diabetic Cataract: A Preliminary Study

David L Williams

Table 1. Signalment of dogs, serum fructosamine and duration cataract-free.

Case	Treatment	Breed	Gender	Age	Serum Fructosamine	Duration Cataract-Free
1	placebo	dachshund	fn	11	380	184
2	placebo	labrador x	mn	9	425	180
3	placebo	labrador x	me	8.7	383	112
4	placebo	labrador	mn	11	458	106
5	placebo	cross-bred	me	12	451	108
6	placebo	cross -bred	fn	9.9	446	180
7	placebo	minature poodle	fn	13	688	220
8	placebo	dobermann	fn	9	465	240
9	placebo	WHWT	mn	8	356	148
10	placebo	collie cross	fn	9	497	139
11	placebo	WHWT	fn	10.3	547	210
12	placebo	beagle	me	12	768	260
13	placebo	JRT	me	10.9	657	245
14	placebo	border collie	mn	9	556	235
15	placebo	cross-bred	fn	10.9	634	260
16	ALA	dachshund	fn	10	463	240
17	ALA	dachshund	fn	9.5	536	265
18	ALA	terrier cross	mn	9	523	215
19	ALA	labrador	mn	8	437	275
20	ALA	labrador	fn	8	367	220
21	ALA	Cavalier king Charles spaniel	fn	7	426	282
22	ALA	Staffordshire bull terrier	fn	11	710	180
23	ALA	Shitzu	mn	9	536	157
24	ALA	lhasa apso	Fn	10.5	635	165

Table S1. *Cont.*

Case	Treatment	Breed	Gender	Age	Serum Fructosamine	Duration Cataract-Free
25	ALA	English bull terrier	Fn	8	734	160
26	ALA	cross-bred	Mn	9	365	220
27	ALA	English springer spaniel	fn	8	462	150
28	ALA	Cross-bred	Mn	8.5	634	178
29	ALA	West highland white terrier	Fn	9	424	175
30	ALA	Border collie	fn	11	318	220

Black indicates no cataract at end timepoint, red indicates cataract developed at end timepoint.

© 2017 by the author; licensee MDPI, Basel, Switzerland. This article is an open access article distributed under the terms and conditions of the Creative Commons by Attribution (CC-BY) license (http://creativecommons.org/licenses/by/4.0/).
